# Metabolomic spectra for phenotypic prediction of malting quality in spring barley

**DOI:** 10.1038/s41598-022-12028-4

**Published:** 2022-05-12

**Authors:** Xiangyu Guo, Ahmed Jahoor, Just Jensen, Pernille Sarup

**Affiliations:** 1 Center for Quantitative Genetics and Genomics, Aarhus University, 8830 Tjele, Denmark; 2 Danish Pig Research Centre, Danish Agriculture and Food Council, 1609 Copenhagen V, Denmark; 3 Nordic Seed A/S, 8300 Odder, Denmark; 4 Department of Plant Breeding, The Swedish University of Agricultural Sciences, 2353 Alnarp, Sweden

**Keywords:** Metabolomics, Plant breeding

## Abstract

We investigated prediction of malting quality (MQ) phenotypes in different locations using metabolomic spectra, and compared the prediction ability of different models, and training population (TP) sizes. Data of five MQ traits was measured on 2667 individual plots of 564 malting spring barley lines from three years and two locations. A total of 24,018 metabolomic features (MFs) were measured on each wort sample. Two statistical models were used, a metabolomic best linear unbiased prediction (MBLUP) and a partial least squares regression (PLSR). Predictive ability within location and across locations were compared using cross-validation methods. For all traits, more than 90% of the total variance in MQ traits could be explained by MFs. The prediction accuracy increased with increasing TP size and stabilized when the TP size reached 1000. The optimal number of components considered in the PLSR models was 20. The accuracy using leave-one-line-out cross-validation ranged from 0.722 to 0.865 and using leave-one-location-out cross-validation from 0.517 to 0.817. In conclusion, the prediction accuracy of metabolomic prediction of MQ traits using MFs was high and MBLUP is better than PLSR if the training population is larger than 100. The results have significant implications for practical barley breeding for malting quality.

## Introduction

Brewing for alcohol production is the major end use of malt, and barley is the primary cereal used in production of malt because of optimal content of carbohydrates, dietary fibers, protein, vitamins and minerals^1^. In the process of brewing, the cereal needs to be malted. Under specific controlled conditions, cereal grains are sprouted and the young seedlings are grown for four to six days in order to produce malt^2^. This process ensure a physical and biochemical transformation within the grain that is defined as malting^1^. During the process of malting, the cell-wall is degraded and protein is broken-down by hydrolytic enzymes such that the physical and the biochemical structure of the barley grain is modified in order to allow malt to be used in the subsequent stages of brewing^3^.

The quality of malt can directly affect the quality and quantity of brewed beer, thus malting quality (MQ) traits are important traits in breeding of barley to be used for malting. A series of MQ traits are defined in the malting industry. These include traits as extract yield and grain protein; alpha-amylase, beta-glucanase, beta-glucan, soluble protein, and free amino nitrogen in wort; and some physical properties such as diastatic power, viscosity, taste, flavor, haze and foam head retention^4^. Measurement of MQ traits is expensive and labor-intensive and the MQ traits have been demonstrated to have complex inheritance^5,6^. A detailed analysis of genetic variation in MQ traits in spring barley was provided by a previous study^6^, where a population of 1329 spring barley lines from four breeding cycles were investigated and medium to high narrow sense heritabilities were found for the MQ traits included in this study.

The organic compounds in a plant are mostly produced by the plant itself so that the photosynthetic and metabolic capacity of a plant is the primary factor determining its growth potential^7^. Metabolites are typical intermediates of biochemical reactions during the growth and development at all stages of plant life^8^. A comprehensive view of cellular metabolites can be provided by metabolomics, which is an approach to quantify the endogenous metabolites in cells and organisms. The development of metabolomics has contributed to the molecular and biological characterization of various organisms. Especially in the area of crops, compared with animals and microorganisms, metabolomics is of great importance since the crops produce very large array of metabolites collectively^9^. Omics technologies like genomics, transcriptomics, and metabolomics can be used in the investigation for the biological background in different organisms^10^.

Nuclear magnetic resonance (NMR) spectroscopy is one of the technologies used to analyze many metabolites simultaneously^11^. NMR can produce signal intensities, which can be treated as an indicator of metabolites in a biological sample, were defined as metabolomic features (MFs)^12^. A total of 24,018 MFs from barley wort were investigated in a previous study where the genetic variation in the MFs was investigated using a univariate model and 8,604 MFs were found to be significantly heritable^13^. NMR has been recognized as one of the most powerful analytical techniques which allows detailed investigation of qualitative and quantitative characteristics of complex chemical and biological samples^14^.

The most popular regression method in the field of chemometrics is partial least squares regression (PLSR)^15,16^. PLSR was first developed for the modelling of information-scarce situations in social sciences by Wold^17^. It is a latent variable approach which has been used to find fundamental relationships between two matrices by modelling the inner covariance structures^18^. Similar with traditional regression, PLSR relates two matrices by a linear multivariate model, but compared with traditional regression, the structure of two matrices can also be modelled when using PLSR^19^. The use of PLSR in chemistry first started in 1980s and has increased steadily for about 40 years, due to its appealing mathematical properties^20^. PLSR is able to analyze data sets with a large number of explanatory variables compared to the number of observations, in cases of noisy data, multi-collinearity, and incomplete variables in both the matrix of dependent variables and the matrix of predictor variables^19,21^.

Best linear unbiased prediction (BLUP), which is a method allowing prediction of random effects in a mixed model, was originally developed in animal breeding for prediction of breeding values (BVs) ^22^. In the area of animal breeding, the selection of animals with highest BV was usually based on predicted/expected BVs (EBVs) derived from the records on the animals themselves and their relatives using BLUP. The use of BLUP is also widely studied in many other areas of research where the use of mixed linear models are relevant such as plant breeding^23^.

BLUP can be used in general linear mixed models that include both fixed and random effects. The simplest case is BLUP without pedigree, where genotypic effect is treated as an independent unobservable normally distributed random variable and no relationships between individuals are considered^23^. Compared with a model based on individual performance, pedigree based BLUP leads to more accurate predictions and result in larger genetic gain because it efficiently uses information from all relatives by constructing an additive genetic relationship matrix (**A**), under the circumstances where genetic relationships between relatives exists^24^. The higher the additive genetic relationship between the genotype of interest with its relatives, the more information can be gained from records of these related genotypes^23^. With the rapid development of biochip technology, genomic BLUP (GBLUP) has been developed and widely applied because it is easy and straightforward to be implemented since technically it just needed the replacement of the **A** matrix in pedigreed based BLUP by a genomic matrix (**G**)^25^. More recently, metabolomic BLUP (MBLUP) has been proposed by replacing the **A** or **G** matrix by a metabolomic similarity matrix (**M**) and MBLUP has been shown as an promising method^26^.

In our previous study, around 36% of MFs were found having significant heritability and among which many were found to be correlated with MQ traits in spring barley^13^. With this information, it is worthwhile to investigate the role of MFs involved in the prediction of phenotypes for MQ traits. PLSR is a popular method used in the studies of metabolic profiles^27^, and using metabolomic BLUP (MBLUP) model gave better prediction accuracies than the BLUP model using genomic information for four of five quantitative traits investigated^28^.

The objectives of this study were to: (1) investigate the possibility of prediction of phenotypes of malting quality traits using metabolomic information; (2) compare the ability of predictions using PLSR and MBLUP models; (3) study the effect of different training population size on the accuracy of prediction; (4) explore the possibility of metabolomic prediction within and across location; and (5) compare different number of components considered in the PLSR model.

## Materials and methods

All the data used are available in a public accessible repository^29^.

### Field trials

In this study, a total of 2667 plots of 564 spring barley malting lines were included. These lines were part of the standard breeding program from Nordic Seed A/S. All experiments in this study were conducted on land owned by Nordic Seed. There were no animal or human experiments conducted for this research, the study also did not contain any GMO. Standard farm operating procedures were used and therefore no ethical approval was needed for this study. All the experiments involving plants adhered to plant ethics guidelines. Samples from two locations in Denmark were used, and samples were taken from each plot individually and the data covered three years from 2014 to 2016. The two locations are Dyngby (55° 56′ 57.2″ N 10° 15′ 13.8″ E) and Holeby (54° 42′ 03.1″ N 11° 27′ 07.6″ E). In both locations, the fields were divided into trials, which included 52–106 plots (8.25 m^2^). Each trial was designed as a randomized complete block comprising 20–45 lines with three replicates of each line^30^. The capacity of trials varied so that 564 lines were not exactly equally distributed into all the trials. The breeders reference of the lines involved in this study was not provided in the published repository^29^ in order to comply with business rules. Each trial included two control lines in three replications. As a consequence, testing was conducted in a number of trials within each year-location combination. In total there were 139, 214 and 215 lines tested in 2014, 2015, and 2016, respectively. Two lines were tested in all three years as standards and the 2667 plots were distributed on 564 inbred lines.

### Measurements of malting quality traits

Malt sample from each plot was milled and extracted in water in order to produce a wort as described in the previous study^13^. The wort was used to measure five MQ traits which included filtering speed (FS), extract yield (EY), wort color (WC), beta glucan content (BG), and wort viscosity (WV). The wort samples needed to be filtered first, and 20 min after filtering begun, FS was scored by measuring the height of the liquid surface in the glass (cm flow-through in 20 min). EY was the percentage of dry matter in the filtered wort. Spectrophotometer was used to determine WC following the method of European Brewery Convention (EBC)^31^. After the filtration, the wort samples were separated in two parts and all wort phenotypes were obtained according to the Analytica-EBC 2004 manual. Briefly, one sample of 25 ml of wort was used for WV (mPa/s, Analytical-EBC 8.4) and EY (Analytical-EBC 8.3). A second sample of 3–4 ml of wort was used for BG (mg/l, Analytical-EBC 8.13.1) and WC (Analytical-EBC 8.5). Detailed description of MQ traits also can be found in a previous study by Sarup, et al.^6^, where the heritability estimates for MQ traits were reported as 0.51 for EY, 0.31 for FS, 0.64 for WC, 0.55 for BG, 0.49 for WV.

### Metabolomic features and NMR intensities

The preparation of NMR analysis is described in detail in Guo, et al.^13^. MFs used in this study were 24,018 NMR intensities which obtained from one-dimensional (1D) ^1^H NMR spectra. The NMR intensities were integrated over small chemical shift (δ) intervals and expressed in parts per million (ppm) in the frequency range of 0.00–11.00 ppm. An in-house custom Matlab script was used to process the spectra^32^. ﻿First an exponential apodization function equivalent to 0.5 Hz line-broadening was used and then Fourier transformation was applied. Afterwards, all spectra were referenced to the DSS-d_6_ signal, automatically phased, and baseline corrected. After visual inspection data below 0.70 ppm and above 9.00 ppm was removed as it did not contain any signal. The water peak which was in the range of 4.7–4.9 ppm, and the region of the added standard which was − 0.2 ppm to 0.2 ppm, were also excluded. The raw data was then normalized using the probabilistic quotient method^33^, and the spectra were aligned using icoshift^34,35^. Finally, the MFs were centered and standardized to a mean of 0 and standard deviation as 1 in order to equalize the contribution from each observation independent of signal intensity^12^.

### Statistical models and methods

Two models were involved in the statistical analyses. The models were a metabolomic best linear unbiased prediction (MBLUP) model and a partial least squares regression (PLSR) model.

### Metabolomic best linear unbiased prediction (MBLUP)

MBLUP model was as follows:

$${\varvec{y}} = 1{\varvec{\mu}} + {\varvec{m}} + {\varvec{e}},$$

where ***y*** referred to the vector of each MQ trait, ${\varvec{\mu}}$ was intercept, ***m*** was the vector metabolomic effects, and ***e*** was a vector of residual terms that could not be explained by the other effects in the model. In this model, ${\varvec{\mu}}$ was a fixed parameter, ***m*** was a random vector with ${\varvec{m}}\sim N\left( {0,{\varvec{M}}\sigma_{m}^{2} } \right)$, and ***e*** was a random vector with ${\varvec{e}}\sim N\left( {0,{\varvec{I}}\sigma_{e}^{2} } \right)$. The reason for only ${\varvec{\mu}}$ taken as fixed parameter, instead of following the model in our previous study to consider more fixed parameters^13^, was because metabolomic information include environmental factors in addition to genomic information. **M** denoted the metabolomic similarity matrix built from MFs using the method as for building a genomic relationship matrix (**G**) computed using VanRaden method 1^25^. Specifically, ${\varvec{M}} = \frac{{\user2{QQ^{\prime}}}}{m}$, where **Q** is a n × m matrix of adjusted, centered and scaled NMR intensities with m = 24,018 (equal to number of MFs) and n = 2667 (equal to number of samples). Both the build of **M** and the MBLUP analysis were carried out by using the “qgg” R-package^36^.

The (co)variance components in the MBLUP model described in the previous section were estimated by restricted maximum likelihood using “qgg” R-package^36^.

The total variation of each MQ trait was calculated as the sum of variance components in MBLUP model:

$$\sigma_{P}^{2} = \overline{M}\sigma_{m}^{2} + \sigma_{e}^{2} ,$$

where $\overline{M}$ was the average diagonal of **M**, which equal to 1. The relative variance component due to effects of ***m*** was $RVC_{m} = \frac{{\overline{M}\sigma_{m}^{2} }}{{\sigma_{P}^{2} }}$ which describe the proportion of total variation (across fixed effects) in MQ traits that can be described by the MFs.

### Partial least squares regression (PLSR)

The PLSR model decompose **Q**, the matrix of MFs, into orthogonal scores **T** and loadings **P**:

$${\varvec{Q}} = {\varvec{TP}},$$

so that regressing ***y*** not on **Q** itself but on the first *t* columns of the scores ***T***, *t* is the number of components fitted in the model when using the package mentioned below. The number of principal component (PCs) were 2667 which was the number of observations in the metabolomic data. PC1 explained large proportion of variance in MFs and 99.99% of variance in MFs is explained by first 20 PCs as shown in Figure S2. Detailed description of PLSR method can be found in the documentation of the “pls” package^21^, which was used in the current study to carry out the PLS analysis.

### Cross-validation

Three different leave-set-out (LSO) cross-validation strategies, in which the whole dataset was divided into a training population (TP) and a validation population (VP), were investigated in this study based on three different hypotheses regarding factors that influence prediction accuracies. The first strategy, named SIZE, was to randomly leave out VP in order to create TP of different size; the second strategy, named LINE, was to leave out VP according to line i.e. all observations of a specific line; the third strategy, named LOC, was to leave out VP according to location.

In the SIZE strategy, since the TP samples and VP samples were randomly selected, the TP contained observations on the same lines, locations and years as in VP—although not the same combinations of lines, locations and years. In the LINE strategy, one out of 564 lines was left out so that the accuracy of predicting one line from all the other lines could be investigated, this strategy is similar to prediction of new lines. In the LOC strategy, one out of two locations was left out, which means the accuracy of predicting one location based on data from the other one location could be investigated.

For each strategy, cross-validation was carried out to evaluate the accuracy of metabolomic prediction of five MQ traits using MFs. In order to study the effect of different size of TP in SIZE strategy, eight scenarios were investigated in this study. These scenarios varied on TP having the size of 50, 100, 200, 500, 1000, 1500, 2000, and 2500. Since the selection of TP was random, 15 replicates were carried out when selecting the TP. For the strategy of LINE, data from 564 lines were left out separately so that 564 replicates were carried out in this strategy. For the strategy of LOC, data from one of the two locations were left in turn so each location were predicted based on the other location.

In each round of the cross-validation, according to the setup of TP size, a certain number of the phenotypes were selected and then the rest of phenotypes were masked. The phenotypes of the masked samples were predicted based on the TP together with the metabolomic information. Thereafter, the correlation between phenotypes and the predicted values was calculated as the accuracy of prediction. The accuracies obtained from strategy LINE and LOC were computed based on both plot level and line mean level where means were computed both for MQ and predicted MQ. This means, two accuracies were obtained for each model in LINE and LOC strategies. In each round of the prediction in LINE and LOC strategies, the predicted values for VP in this round were collected, and then when all the rounds of prediction completed, predicted values were collected for the whole population. Afterwards, the accuracy on plot level in LINE and LOC strategies was calculated as the correlation between observed phenotypes and the predicted values of each plot, and the accuracy of line mean was calculated as correlation between average observed phenotypes and the average predicted values of each line.

When applying PLSR model, compared with MBLUP model, a leave-one-sample-out (LOO) cross-validation was carried out within the TP to train the model first, and afterwards the LSO cross-validation was processed using the trained model from the preliminary LOO cross-validation. In this study, different number of components considered in the PLSR model was also compared, and the number of components were 5, 10, 20 and 50. The cumulated proportion of MF variance explained by first 60 components are shown in Figure S2.

### Software and setup

The cross-validation procedure using MBLUP was carried out by “qgg” package^36^ and the procedure using PLSR model was carried out by “pls” package^21^. In all three strategies, both MBLUP and PLSR models were applied, and the datasets utilized in each round of the cross-validation were same for MBLUP and PLSR models to make sure they were comparable.

## Results

In this study, the proportion of total phenotypic variance across fixed effects in malting quality (MQ) traits that can be explained by effects of metabolomic features (MFs) was evaluated. Then the phenotype of MQ traits was predicted by using MFs through MBLUP and/or PLSR models. Different size of TP, and the different number of components considered in the PLSR models, prediction across line and location were also investigated.

### Descriptive statistics for malting quality traits

Table 1 gives descriptive statistics of all the MQ traits analyzed in this study. There were 2667 records analyzed for five MQ traits. The average of all the traits were 4.83 for FS, 82.66 for EY, 5.83 for WC, 217.10 for BG, and 1.47 for WV. The coefficient of phenotypic variance ranged from 2.21% for EY to 53.07% for BG.

**Table 1:** Descriptive statistics for malting quality traits.

Trait	No. of records	Unit	Average	SD	Min	Max	CV (%)
FS	2667	cm/20 min	4.83	0.61	2.30	6.30	12.72
EY	2667	%	82.66	1.82	70.38	92.39	2.21
WC	2667	EBC units	5.83	0.83	3.59	8.99	14.23
BG	2667	mg/L	217.10	115.23	70.00	751.19	53.07
WV	2667	mPa s	1.47	0.06	1.29	1.73	4.27

Trait: FS = filtering speed, EY = extract yield, WC = wort color, BG = beta glucan, WV = wort viscosity; CV is coefficient of phenotypic variance.

### Estimates of total variance of malting quality traits explained by metabolomic features

A univariate MBLUP model was applied to estimate the proportion of the total variance including potential fixed effects in each MQ trait that can be explained by the MFs. The estimates were indicators for the proportion of total variance in MQ traits that is associated with metabolites.

**Figure 1:**
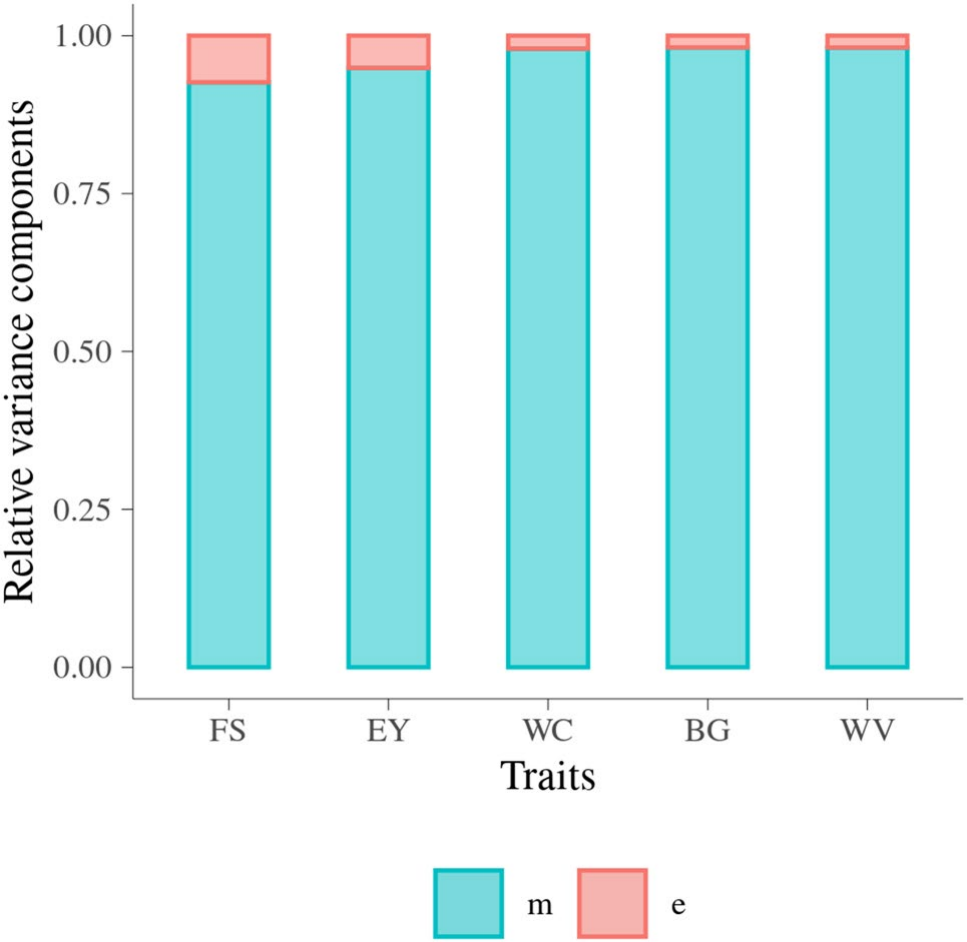
Proportion of total variance explained by metabolomic features and error in malting quality traits. Trait: FS = filtering speed, EY = extract yield, WC = wort color, BG = beta glucan, WV = wort viscosity; y-axis is relative variance component; m is relative variance of metabolomic effects and e is relative variance of residuals.

Figure 1 shows the estimated relative amount of total variance explained by MFs (RVCm) and error in five MQ traits. The RVCm ranged from 0.93 ± 0.01 in FS to 0.98 ± 0.00 in BG.

For all the MQ traits, the effect of MFs explained very large proportions of the total variance. RVCm in WC, BG and WV were similar and larger than RVCm in FS and EY.

### Metabolomic prediction using MBLUP model

**Figure 2:**
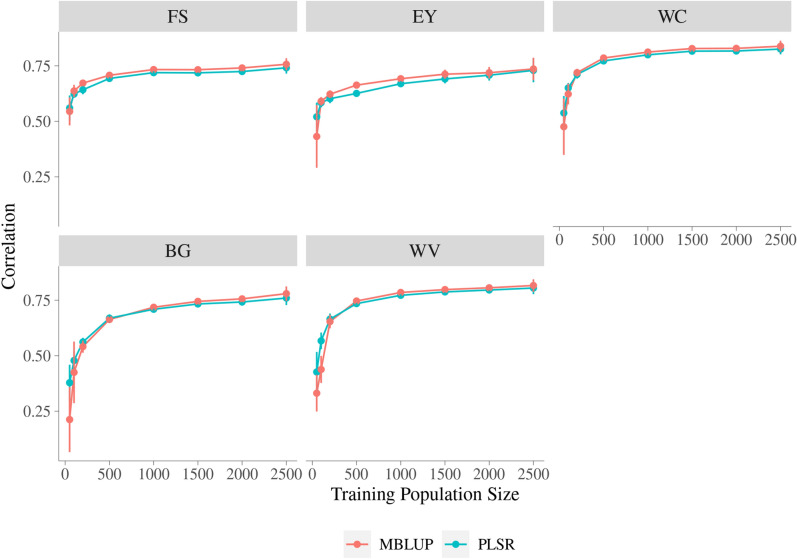
Accuracy of prediction for malting quality traits using MBLUP and PLSR models with different training population size. Trait: FS = filtering speed, EY = extract yield, WC = wort color, BG = beta glucan, WV = wort viscosity; x-axis is training population size, y-axis is accuracy of prediction which is the correlation between observed and predicted phenotypes; MBLUP is metabolomic best linear unbiased prediction model, PLSR is partial least squares regression model; PLSR at each point are the results from PLSR model with best number of components.

A univariate MBLUP model was used for metabolomic prediction of the five MQ traits. The cross-validation results from MBLUP model at each SIZE scenario are shown in Fig. 2. As shown in Fig. 2, averaged across 15 replicates in the strategy of SIZE, the maximum prediction accuracies using MBLUP model were 0.76 ± 0.03 for FS, 0.74 ± 0.05 for EY, 0.84 ± 0.03 for WC, 0.78 ± 0.03 for BG and 0.82 ± 0.03 for WV. In addition, for all traits, the maximum accuracy were obtained in the scenarios with 2500 as TP size.

### Metabolomic prediction using PLSR model

**Figure 3:**
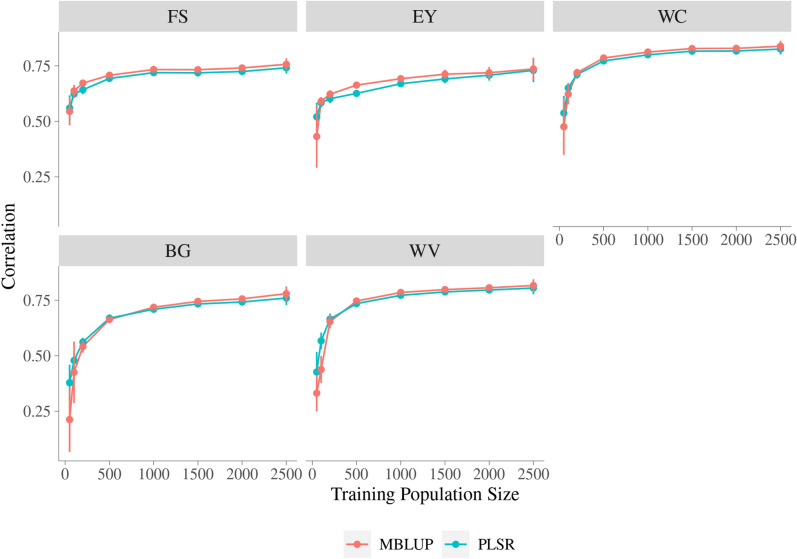
Accuracy of prediction for malting quality traits using PLSR models with different training population size. Trait: FS = filtering speed, EY = extract yield, WC = wort color, BG = beta glucan, WV = wort viscosity; x-axis is training population size, y-axis is accuracy of prediction which is the correlation between observed and predicted phenotypes; PLSR is partial least squares regression model; PLSR_05–PLSR_50 are partial least squares regression models with different number of components (5, 10, 20, 50).

There were four PLSR models compared regarding the number of components utilized in the model. The number of components considered were 5, 10, 20 and 50. As shown in Fig. 3, averaged across 15 replicates, the maximum prediction accuracies using PLSR model were 0.74 ± 0.03 for FS, 0.73 ± 0.05 for EY, 0.83 ± 0.02 for WC, 0.76 ± 0.03 for BG and 0.81 ± 0.03 for WV. In addition, all the maximum accuracy were obtained when using PLSR model with 20 components, except FS, for which the maximum accuracy was provided by the PLSR model with 10 components, but it was very close to the accuracy provided by PLSR model with 20 components.

When the PLSR model considered 5 components, the prediction accuracy was low for all the MQ traits. With increase in the number of components considered in the PLSR model, the accuracy also generally increased. The accuracy kept increasing until the number of components reached 20. However, when the number of components increased further to 50, the accuracy decreased and in some cases even smaller than the accuracy from 5 components.

### Comparison of MBLUP and PLSR models

The accuracy from MBLUP and the maximum accuracy among four PLSR models are plotted for each SIZE scenario in Fig. 2. The accuracy obtained from MBLUP model was smaller than at the maximum accuracy from PLSR model when the TP size was small. When the TP size was 50, MBLUP yielded smaller accuracy in all the five MQ traits. With the increase of TP size, the accuracy from MBLUP increased rapidly and was larger than for the PLSR model. For example, in FS, MBLUP yielded higher accuracy than PLSR when the TP size just increased to 100. For all the traits, MBLUP yielded higher or same accuracy compared with all the PLSR models when the TP size reached 500.

### Metabolomic prediction with different training population size

A total of eight sizes, including 50, 100, 200, 500, 1000, 1500, 2000, and 2500, of TP were compared. With the increase of TP size, the prediction accuracy increased regardless of the model used. When the TP size was small, PLSR could provide better predictions than MBLUP model, while along with the increase of TP size, the MBLUP outperformed PLSR models as soon as the data size reached 500 samples. As can be observed from Fig. 2, though the general trend was higher accuracy obtained in the scenario of largest TP size. When the TP was increased beyond 1000, the increase in accuracy was limited.

### Metabolomic prediction of new lines

**Figure 4:**
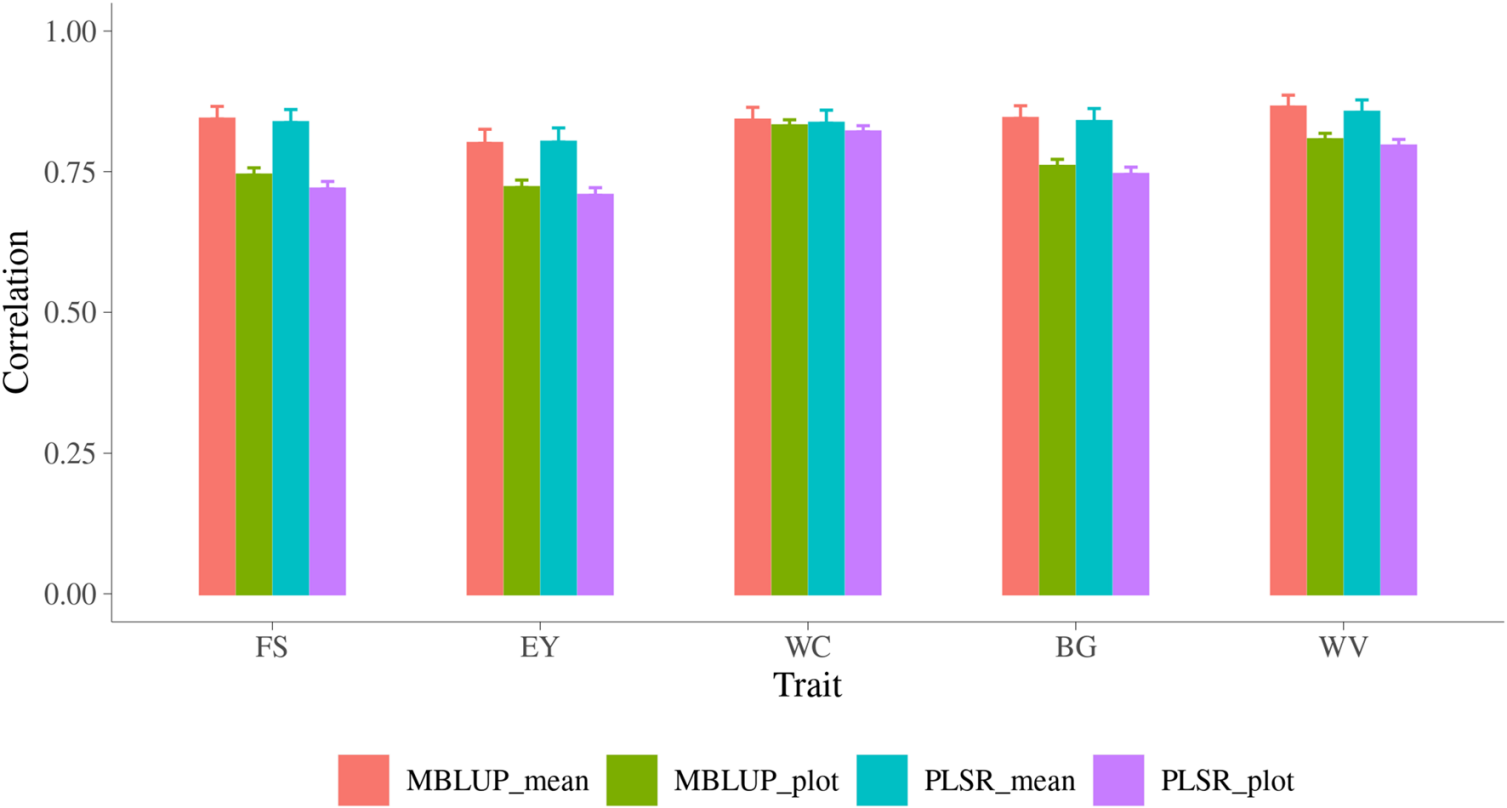
Accuracy of prediction for malting quality traits across line using MBLUP and PLSR models. Trait: FS = filtering speed, EY = extract yield, WC = wort color, BG = beta glucan, WV = wort viscosity; y-axis is accuracy of prediction which is the correlation between observed and predicted phenotypes; MBLUP is metabolomic best linear unbiased prediction model; PLSR is partial least squares regression model with 20 components; plot is accuracy of plot, mean is accuracy of line mean.

The second cross-validation strategy investigated in this study was LINE, in which the data from one line were masked as VP and the data from the other lines were treated as TP to predict the VP. This corresponds to predicting a new line based on metabolomic information only. There were 564 lines in the whole dataset, one line was left out and then predicted based on all other lines. This process was repeated until all lines were predicted. Since PLSR model with 20 components generally yielded highest accuracy, only this PLSR model was conducted and compared with MBLUP model in this strategy. As shown in Fig. 4, when using MBLUP model, the accuracy of plot ranged from 0.72 ± 0.01 for EY to 0.83 ± 0.01 for WC, and the accuracy of line mean ranged from 0.80 ± 0.03 for EY to 0.87 ± 0.02 for WV. The MBLUP surpassed PLSR model though the difference are small.

### Metabolomic prediction across location

**Figure 5:**
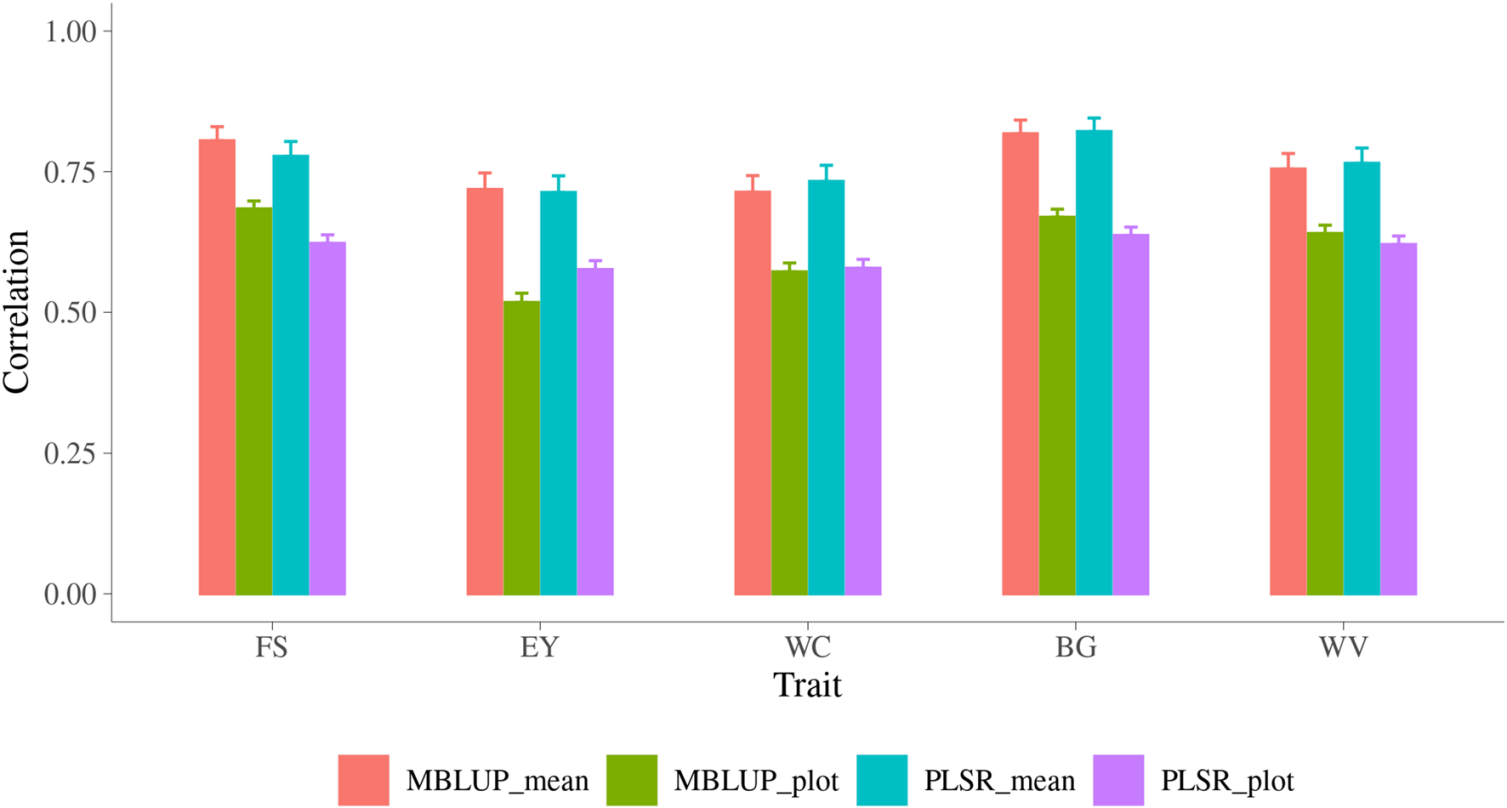
Accuracy of prediction for malting quality traits across location using MBLUP and PLSR models. Trait: FS = filtering speed, EY = extract yield, WC = wort color, BG = beta glucan, WV = wort viscosity; y-axis is accuracy of prediction which is the correlation between observed and predicted phenotypes; MBLUP is metabolomic best linear unbiased prediction model; PLSR is partial least squares regression model with 20 components; plot is accuracy of plot, mean is accuracy of line mean.

The third cross-validation strategy investigated in this study was LOC, in which the data from one location were masked as VP and the data from the other location were treated as TP to predict the VP. There were two locations in the whole dataset, therefore, this strategy had two rounds of prediction by treating each location as VP in each round. Same with LINE strategy, both MBLUP and the PLSR with 20 components were carried out in this strategy. As shown in Fig. 5, the accuracy of plot ranged from 0.52 ± 0.02 for EY to 0.68 ± 0.01 for FS, the accuracy of line mean ranged from 0.71 ± 0.03 for WC to 0.82 ± 0.02 for BG, when using MBLUP model. The accuracy provided by PLSR model were similar with MBLUP.

## Discussion

Metabolomic prediction using 24,018 metabolomic features (MFs) were carried for a total of 2667 plots of 564 spring barley malting lines each phenotyped for five malting quality (MQ) traits. MBLUP and PLSR models were compared. Accuracy of cross-validation was investigated by varying size of training population, also using leave-one-line-out and leave-one-location-out strategies. In addition, the number of components in the PLSR model was also studied.

### Descriptive statistics for malting quality traits

The descriptive statistics for MQ traits in the current study were similar with the previous study though the number of observations in the previous study was around three times larger than in the current study^6^. The standard deviation of most of the MQ traits in the current study were smaller than the previous study and it is expected because in the previous study, 1,329 spring malting barley lines were involved and the harvest was done in four different years and three locations^6^, so that the samples from the current study was a subset of the previous study. The more variation in the year and location compared with the current led to the larger variation in phenotypes.

### Estimates of total variance of malting quality traits explained by metabolomic features

The variance of five MQ traits explained by MFs was explored by using a univariate model integrating a metabolomic similarity matrix and the proportion of metabolomic effects were larger than 90% for all the five traits.

The utilization of metabolomic similarity matrix in the model aimed at dissection of the total variance into a metabolomic part and a random error. The proportion of the variance of MFs shows the extent that MFs can be used to predict total variance in MQ traits. In our previous study, WC, BG, and WV were found to have significant phenotypic and genetic correlation to a large proportion of the MFs^13^, similarly to a large extent, their total variance could be explained by MFs. Potentially MQ traits of WC, WV, and BG can be predicted from the MFs because MFs explains almost all the variance in these MQ traits. While among the two traits (FS and EY) having relative lower correlation with MFs, they could not be explained to the same very high degree by variation in metabolites. One of the reasons that there was a very large proportion of total variance in BG could be explained by MFs, can be due to that BG itself is a metabolite included in the NMR peaks.

The direct link between metabolites and phenotypic records in biological systems provide the potential of utilizing metabolomic features as an objective proxy for phenotype data^37^. The fact that almost all the variation in MQ can be explained by the MFs confirmed that the MFs could be used as the objective proxy for phenotype of interest and even more valuable and meaningful when the phenotype of interest is difficult or expensive to be obtain.

### Metabolomic prediction using MBLUP model

The MBLUP model used for estimation of variance components was then used for metabolomic prediction of five MQ traits. The prediction accuracies using MBLUP model were quite promising as the maximum accuracy were all above 0.7. This is higher than the previous reported prediction accuracies for metabolomic prediction of plant phenotypes^7,38–41^. The higher prediction accuracy in this study is probably due to a larger number of unique genotypes in the study and the fact that the NMR was performed directly on wort and not on, for example, leaves of the developing plant. When utilizing genomic information and fitting a genomic BLUP (GBLUP) model, the accuracies of genomic prediction for MQ traits were reported as from 0.28 to 0.68^6^. The accuracy of GBLUP in the previous study was lower than the accuracy of MBLUP in the current study can be due to metabolomic data included information on both genetic factors as well as environmental factors. Thus the metabolomic information was closer related with phenotype observations than the genomic information. Though the spring barley lines involved in the current study were from the same breeding program as the lines in the previous study^6^, the number of lines studied in the current study was a subset from the previous one, which can also lead to the difference in prediction accuracy. However the accuracy of GBLUP is expected to be even lower if using the same dataset as in the current study, which is smaller than and be a subset of the dataset in the previous study^6^.

One of the reasons for the high prediction accuracy using MBLUP could be because the total variance explained by MFs were large in all the five MQ traits. The phenotypes of MQ traits can be predicted very well and better than when using GBLUP, because most variation in MQ is expected to be reflected in the NMR spectra. The high accuracy also shows that there is no overfitting and MBLUP can explain and predict large part of the variation in MQ. In addition, our previous study on the genetic and phenotypic correlation between MFs and MQ traits also showed a significant correlation between them, which can also be the reason for the high prediction accuracy using metabolomic information^13^. A subset of MFs were detected as significantly heritable, and a further subset of these had significant genetic correlation with MQ traits in our previous study^13^.Therefore, we carried out extra analysis in order to compare the performance of MBLUP using different subsets of MFs. Three matrices were built regarding to MFs included, the matrix using all the 24,018 MFs was M, the matrix using significant heritable MFs was Ms, and the matrix using MFs which were significant heritable and also had significant genetic correlation with each trait was Mgs (varied across traits). The estimation of variance due to MFs were quite similar among the MBLUP models using M, Ms and Mgs. The accuracy of prediction for using these three MBLUP models with different training population size are shown in the Supplementary Figure S1. Very similar results obtained from three MBLUP models (M, Mgs, Ms) indicated that selecting the significant heritable MFs and/or MFs having significant genetic correlation with traits did not improve the prediction accuracy of MBLUP. When applying MBLUP in the breeding system, breeders can directly utilize all the MFs instead of filtering out some part of MFs which may involve more work, cost, and potential for errors.

The performance of GBLUP and MBLUP was investigated in *Drosophila*, where the prediction accuracy for two behavioral traits was below 0.1 when based on GBLUP and then increased to above 0.4 when using MBLUP. Such an increase have also been found for two environmental stress resistance traits in *Drosophila*^28^. In the plant field, metabolic information was introduced into prediction of complex traits by Riedelsheimer, et al.^38^, where the authors presented a complementary approach to exploit large-scale genomic and metabolic information in hybrid testcrosses. The MBLUP was also investigated in a previous study^42^, where metabolomics data were used to predict the performance agronomic traits in wheat, and metabolomic information were found as providing strong predictive power for number of grains per spike and plant height^42^.

### Metabolomic prediction using PLSR model

Four PLSR models were compared with different the number of components considered in the model. The maximum accuracies provided by PLSR models were smaller than for the MBLUP model. Increasing the number of components considered in the PLSR models generally led to the increase of prediction accuracy while when the number of components reached 50, the prediction accuracy was not larger than the one provided by the models only considered 20 components. These results indicated that the non-linear relationship between the number of components and the performance of the prediction. For all the MQ traits investigated in the current study, 20 components were already enough to provide good prediction accuracy though the exact number of components varied a bit from trait to trait. In a study of genomic selection for pork pH traits, 30 was found as the optimal number of components considered in the PLSR analysis^43^.

PLSR has also been suggested as an efficient method to analyze genomic data, because of its ability to handle large data sets and its prediction ability, and the PLSR approach is particularly suitable to predict dependent variables from a very large number of predictors and especially the predictors might be highly correlated with each other^44^. The accuracy of prediction for yield traits in French dairy cattle were similar between PLSR and GBLUP models but in no case PLSR provided higher accuracy than GBLUP^44^. It was also reported that an increase in the number of relevant variables and observations contributed to the improvement in the precision of the model parameters, which was one desirable property of PLSR model^19^.

### Comparison of MBLUP and PLSR models

The comparison of MBLUP and PLSR models showed that MBLUP generally outperformed PLSR for all traits, when the TP size larger than 500. PLSR could be a better choice than MBLUP only when the TP was small. In a previous study Xu et al.^26^ analyzed a hybrid population of rice, and showed that the MBLUP model was superior to PLSR model^26^. A similar situation was also found when utilizing genomic information instead of metabolomic information. For example, a study on rice also investigated the GBLUP model and PLSR using genomic information, showed that the GBLUP outperformed PLSR^26^. The superiority of BLUP model was also found in the study on genomic prediction in French Holstein and Montbéliarde breeds^45^. In addition to the better performance of MBLUP, it is also easy to implement, needs low demands regarding computation power, time and skill for the breeder, which makes MBLUP is more attractive in the practical breeding. For example, the time spent on the analysis of MBLUP when predict phenotypes of one year from the other two years was around 20 s per trait, while was around 5 min when using PLSR model with 20 components. The analysis of MBLUP model was realized by applying public available R package to our data, which can be easily found online together with example codes, which do not need very complicated skills from the breeders.

### Metabolomic prediction with different training population size

A total of eight TP sizes from 50 to 2500 were compared in this study. The results showed that the prediction accuracy generally increased with increasing TP size. Though the accuracy increased all the way from smallest TP until the largest dataset, the increase in accuracy was much smaller when the TP were larger than 1000. The impact of TP size on the prediction accuracy had been demonstrated in the genomic prediction while rare in the metabolomic prediction using metabolomic information^46,47^. For example, the accuracy of genomic prediction in wheat has been investigated regarding to different population sizes and the results indicated that TP of around 700 lines were enough to yield the highest prediction accuracy^48^.

### Metabolomic prediction across line/location

In addition to the first cross-validation strategy which selecting TP randomly within the whole population, two more strategies were investigated either predict the VP from different lines or growing in different locations. The accuracy of predicting plot MQ from these two strategies were smaller than when the TP randomly selected from the whole population. The reason is because when selecting TP from the whole population randomly, the observations on the same lines and/or locations were involved in TP and VP, which increased the degree of the metabolomic similarity between TP and VP.

In this study, 564 lines were harvested in three years separately, which means there was almost no lines involved in two or three years. This design created difficulty in the metabolomic prediction across year based on the current dataset. The across year metabolomic prediction could be better investigated when a dataset including overlap of lines been planted in different years is available.

## Conclusion

Records of five malting quality (MQ) traits and metabolomic features (MFs) for 2667 plots of 564 spring malting barley lines that were grown in two locations were studied. The ability of prediction based on metabolomic information was investigated.

The proportion of variance in MQ traits that can be explained by effects of MFs was above 0.9 for all traits when using all the records. The phenotype of MQ traits could be predicted by MFs through MBLUP and/or PLSR models. The prediction accuracy when using MBLUP was larger than 0.7 and generally surpassed PLSR models when size of training population (TP) larger than 500. When the size of TP smaller than 500, PLSR provided better accuracy than MBLUP. The prediction accuracy increased along with increasing TP size but when the population size reached 1000, the rate of increase was very small. The number of components considered in the PLSR models can affect the performance of PLSR models and 20 was the optimal number. In addition, the prediction accuracy was also explored regarding to using the TP to predict the validation population (VP) in a different year or location. The results showed that it was possible carry out the prediction across line/location with the accuracy of plot ranged from 0.5 to 0.8, and the accuracy of line mean ranged from 0.7 to 0.9.

In conclusion, it is possible to carry out prediction of phenotypes of malting quality traits using metabolomic information. MBLUP is an ideal model for the prediction when TP size larger than 500. The results from the current study indicate that barley breeders can predict MQ based on MFs from the wort and have significant implications for the practical barley breeding.

## Supplementary Information

Supplementary Figures.

## Data Availability

All the data used are available in a public accessible repository with the direct link as https://data.mendeley.com/datasets/s3s4ft92wj/1.

## Abbreviations

BG: Beta glucan
BLUP: Best linear unbiased prediction
BV: Breeding value
CV: Coefficient of phenotypic variance
GBLUP: Genomic best linear unbiased prediction
G: Genomic relationship matrix
EBC: European Brewery Convention
EBV: Expected breeding value
EY: Extract yield
FS: Filtering speed
LOO: Leave-one-sample-out
LSO: Leave-set-out
M: Metabolomic similarity matrix built from all metabolomic features
MBLUP: Metabolomic best linear unbiased prediction
MFs: Metabolomic features
Mgs: Metabolomic similarity matrix built from significant heritable metabolomic features having significant genetic correlation with metabolomic traits
MQ: Malting quality
Ms: Metabolomic similarity matrix built from significant heritable metabolomic features
NMR: Nuclear magnetic resonance
PC: Principal component
PLSR: Partial least squares regression
ppm: Parts per million
RVC: Relative variance component
TP: Training population
VP: Validation population
WC: Wort color
WV: Wort viscosity
