# Supplementary material for: Metabolomic spectra for phenotypic prediction of malting quality in spring barley
Source: Sci Rep. 2022 May 12;12:7881. doi: 10.1038/s41598-022-12028-4 (PMC9098465; doi:10.1038/s41598-022-12028-4)
Supplement: Supplementary file 1 — Supplementary Figures. [file 41598_2022_12028_MOESM1_ESM.docx]

**Metabolomic spectra for phenotypic prediction of malting quality in spring barley**

Xiangyu Guo^1, 2 *^, Ahmed Jahoor^3, 4^, Just Jensen^1^, Pernille Sarup^3^

^1^ Center for Quantitative Genetics and Genomics, Aarhus University, 8830 Tjele, Denmark

^2^ Danish Pig Research Centre, Danish Agriculture & Food Council, 1609 Copenhagen V, Denmark

^3^ Nordic Seed A/S, 8300 Odder, Denmark

^4^ Department of Plant Breeding, The Swedish University of Agricultural Sciences, 2353 Alnarp, Sweden

^*^Correspondance:

Dr. Xiangyu Guo

Xiangyu.Guo@qgg.au.dk

**Supplementary Material**

**
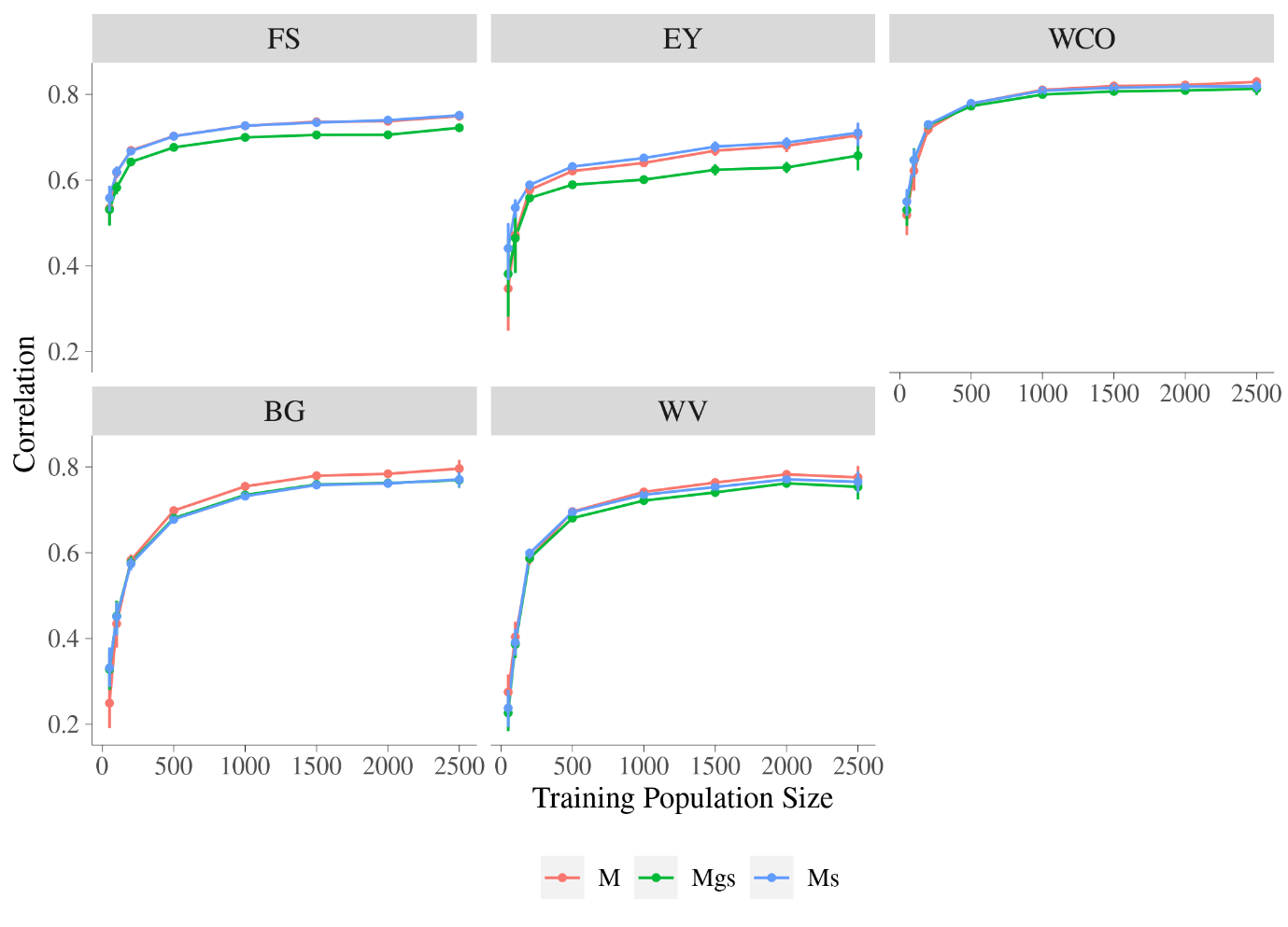
**

## Figure S1 Accuracy of prediction for malting quality traits using MBLUP models with different sets of metabolomic features

Trait: FS = filtering speed, EY = extract yield, WCO = wort color, BG = beta glucan, WV = wort viscosity; x-axis is training population size, y-axis is accuracy of prediction which is the correlation between observed and predicted phenotypes; MBLUP is metabolomic best linear unbiased prediction model, M is MBLUP using all metabolomic features, Mgs is MBLUP using metabolomic features having significant genetic correlation with each trait and significantly heritable, Ms is MBLUP using metabolomic features significant heritable.


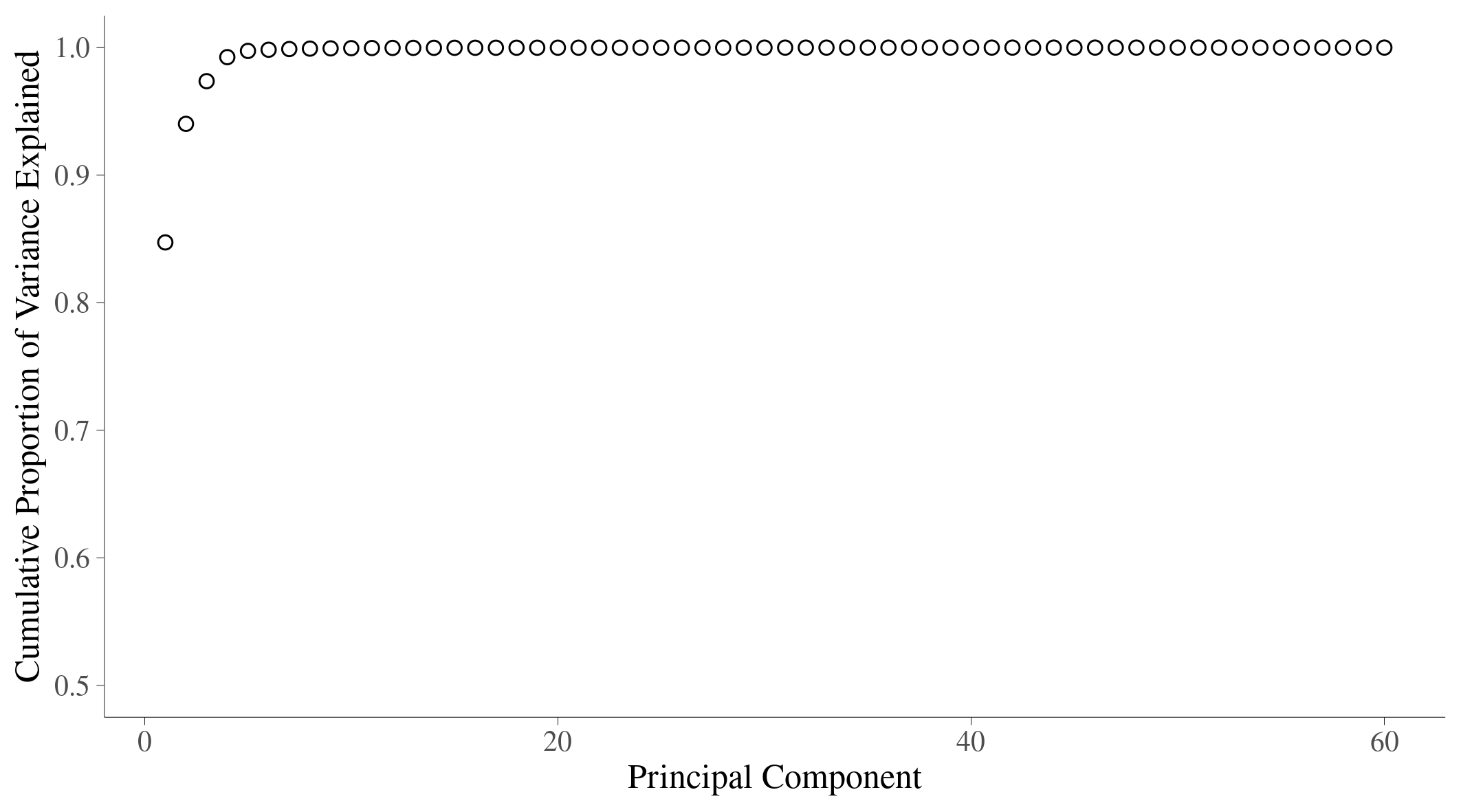


**Figure S2** Proportion of variance explained by principal components in metabolomic similarity matrix

y-axis is the cumulated proportion of variance explained, x-axis is the number of principal components (first 60 components are plotted).
